# Overexpression of Bcl-2 enhances metastatic potential of human bladder cancer cells

**DOI:** 10.1038/sj.bjc.6690264

**Published:** 1999-04

**Authors:** H Miyake, I Hara, K Yamanaka, K Gohji, S Arakawa, S Kamidono

**Affiliations:** Department of Urology, Kobe University School of Medicine, 7-5-1 Kusunoki-cho, Chuo-ku, Kobe, 650, Japan

**Keywords:** Bcl-2, metastasis, apoptosis, bladder cancer

## Abstract

We investigated the effect of Bcl-2 expression on the metastatic process of bladder cancer cells by using the Bcl-2-transfected human bladder cancer cell lines (KoTCC-1/BH) and the control vector only-transfected cell line (KoTCC-1/C), which were generated in our previous study (Miyake et al (1998) *Oncogene* **16**: 933–934). When they were injected intravenously into athymic nude mice, KoTCC-1/BH formed more than three times as many tumour nodules in the lungs as did KoTCC-1/C. In addition, tumour progression, including lymph node metastasis and haemorrhagic ascites, was observed to be more advanced after the implantation of KoTCC-1/BH cells into the bladder wall of nude mice than after implantation of KoTCC-1/C cells. These enhanced malignant progression of KoTCC-1/BH cells were well correlated with anti-apoptotic activity under anchorage-independent conditions in in vitro experimental models. In contrast, there were no significant differences among these cell lines in their growth rates both in vitro and in vivo, invasive ability and cell motility. These findings suggest that, if it is overexpressed, Bcl-2 prolongs cell survival under unfavourable conditions encountered in the metastatic process, resulting in the enhanced metastatic potential of bladder cancer. © 1999 Cancer Research Campaign


*British Journal of Cancer* (1999) **79**(11/12), 1651–1656
© 1999 Cancer Research Campaign
Article no. bjoc.1998.0264

### Overexpression of Bcl-2 enhances metastatic potential of human bladder cancer cells

**H Miyake, I Hara, K Yamanaka, K Gohji, S Arakawa and S Kamidono**

Department of Urology, Kobe University School of Medicine, 7-5-1 Kusunoki-cho, Chuo-ku, Kobe 650, Japan

**Summary** We investigated the effect of Bcl-2 expression on the metastatic process of bladder cancer cells by using the Bcl-2-transfected human bladder cancer cell lines (KoTCC-1/BH) and the control vector only-transfected cell line (KoTCC-1/C), which were generated in our previous study (Miyake et al (1998) *Oncogene* **16**: 933–934). When they were injected intravenously into athymic nude mice, KoTCC-1/BH formed more than three times as many tumour nodules in the lungs as did KoTCC-1/C. In addition, tumour progression, including lymph node metastasis and haemorrhagic ascites, was observed to be more advanced after the implantation of KoTCC-1/BH cells into the bladder wall of nude mice than after implantation of KoTCC-1/C cells. These enhanced malignant progression of KoTCC-1/BH cells were well correlated with anti-apoptotic activity under anchorage-independent conditions in in vitro experimental models. In contrast, there were no significant differences among these cell lines in their growth rates both in vitro and in vivo, invasive ability and cell motility. These findings suggest that, if it is overexpressed, Bcl-2 prolongs cell survival under unfavourable conditions encountered in the metastatic process, resulting in the enhanced metastatic potential of bladder cancer.

**Keywords:** Bcl-2; metastasis; apoptosis; bladder cancer

Bladder cancer is the most common malignancy of the urinary tract, and the fourth or fifth leading cause of cancer-related deaths of men in Western industrialized countries. The prognosis of patients with metastatic bladder cancer is still extremely poor in spite of the recent therapeutic advances, such as improved surgical techniques and perioperative combination chemotherapy (Thrasher and Crawford, 1993). It is thus important to investigate the mechanism of metastasis of bladder cancer, in order to develop novel therapeutic strategies and consequently improve the survival of patients with advanced bladder cancer.

Apoptosis is a crucial event in various physiological processes, such as embryogenesis, organ development and cell proliferation, as well as pathological processes including autoimmune disease and cancer development (Ellis et al, 1991). These phenomena are regulated by several genes and, among them, Bcl-2, initially identified as the proto-oncogene translocated to the immunoglobulin heavy chain locus in human follicular B-cell lymphomas, is recognized as one of the most potent inhibitors of apoptosis induced by a wide variety of stimuli, such as radiation, chemotherapeutic agents and growth factor deprivation (Tsujimoto et al, 1985). Consistently, many investigators have demonstrated that overexpression of Bcl-2 closely correlates with the progression of various human malignancies, including bladder cancer (Gallo et al, 1996; King et al, 1996). Furthermore, recent studies have suggested that prolonged survival of cancer cells may contribute to promote their metastatic potential (Inbal et al, 1997; Shtivelman, 1997; Takaoka et al, 1997).

From these findings, we speculate that Bcl-2 confers a benefit for the progression of bladder cancer through the inhibition of apoptosis induced by a variety of obstacles that cancer cells may confront after detachment from their primary origin; hence, we report here the effect of Bcl-2 expression on the metastatic process of human bladder cancer cells in both in vitro and in vivo experimental models.

*Received 22 April 1998*
*Revised 4 August 1998*
*Accepted 22 September 1998*

*Correspondence to:* H Miyake, Department of Cancer Endocrinology, British Columbia Cancer Agency, 600 West 10th Avenue, Vancouver, British Columbia, V5Z 4E6 Canada

### MATERIALS AND METHODS

### Tumour cell lines

The human bladder cancer cell line KoTCC-1 was established in our laboratory (Miyake et al, 1997), and the Bcl-2-transfected KoTCC-1 cell lines (KoTCC-1/BH1 to KoTCC-1/BH4) and the control vector only-transfected cell line (KoTCC-1/C) were generated by the liposome-mediated gene transfer method in our previous study (Miyake et al, 1998*a*). These cell lines were maintained in RPMI-1640 supplemented with 10% fetal calf serum.

### Northern blot analysis

Total RNA of each cell line was extracted by the acid–guanidinium thiocyanate–phenol–chloroform method. Aliquots of 30 μg of RNA were separated by electrophoresis on 1.2% agarose–formaldehyde gels, capillary-transferred to a nylon membrane overnight, and cross-linked with ultraviolet irradiation. The filters were hybridized at 42°C overnight with a human Bcl-2 cDNA probe labelled with $^{32}$P-deoxycytidinetriphosphate by random primer labelling. The filters were then washed in 0.5% standard saline citrate and 0.1% sodium dodecyl sulphate (SDS)

for 30 min, then for 20 min at 65°C and exposed to X-ray film at – 80°C. After stripping, the membranes were rehybridized with a human glyceraldehyde phosphate dehydrogenase (GAPDH) cDNA probe.

### Cell proliferation assay

To compare the in vitro proliferation of KoTCC-1 sublines, $5 \times 10^3$ cells of each cell line were seeded in each well of 12-well plates, and the number of cells in each cell line was counted daily by triplicate.

### In vitro tumour cell invasion assay

Tumour cell invasion was measured with a membrane invasion culture system (Albini et al, 1987) in a minor modification. Briefly, we used polycarbonate filters with a pore size of 8 μm, coated with varying amounts of basement membrane Matrigel (Becton Dickinson Labware, Lincoln Park, NJ, USA). The coated filters were placed in Boyden chambers, in the upper compartment of which $1 \times 10^5$ cells of each cell line were suspended in Dulbecco's modified Eagle's medium (DMEM/F-12 and in the lower compartment of which fibronectin (25 μg $ml^{-1}$) diluted with DMEM/F-12 was added as a chemoattractant. After 72 h incubation, the filters were fixed in methanol and stained with Giemsa solution. Cells that had migrated to the lower surface of the filters were counted manually at a 200 × magnification and the mean numbers of cells per field in three random fields was recorded. Similarly, cell motility was also assessed by using the Boyden chambers without Matrigel. Each assay was performed in triplicate.

### Limiting dilution assay

The colonization activity and cell viability of KoTCC-1 sublines under solitary cell conditions were evaluated with a limiting dilution assay (Takaoka et al, 1997) in a minor modification. Briefly, $1 \times 10^2$ cells of each cell line were seeded with one cell per well in 96-well microtiter plates and cultured in RPMI-1640 supplemented with 2% fetal calf serum. After 10 days of culture, numbers of appeared colonies were counted. Each assay was performed in triplicate.

### Cell survival assay in suspension

The cell viability of KoTCC-1 sublines under the condition lacking contact with substrate was examined by the previously described method (Nikiforov et al, 1997). Briefly, $5 \times 10^5$ cells of each cell line were suspended in 30 ml of RPMI-1640 supplemented with 2% fetal calf serum, and then added into 50 ml polypropylene tubes. The tubes were subjected to gentle rocking on a rocking platform in a 37°C room in an atmosphere of 5% carbon dioxide for the indicated periods. After completion of the assay, cells were collected from the tubes and replaced in fresh medium onto tissue culture dishes. After 24 h of culture, the cell viability was quantitated by the MTT assay according to a previously reported protocol (Miyake et al, 1998*b*). Each assay was performed in triplicate. In addition, DNA was isolated from each cell line subjected to 24 h floating, and DNA fragmentation analysis was performed as described previously (Miyake et al, 1998*a*).

### Animal studies

Athymic nude mice (Balb/c *nu*/*nu* females, 6–8 weeks old) were purchased from CLEA Japan, Inc. (Tokyo, Japan), and housed in a controlled environment at 22°C on a 12 h light, 12 h dark cycle. Animals were maintained in accordance with the NIH Guide for the Care and Use of Laboratory Animals. Each experimental group consisted of 10 mice. Each of the tumour cell lines was trypsinized, washed twice with phosphate-buffered saline (PBS), and injected subcutaneously with $1 \times 10^6$ cells in the flank, intravenously with $1 \times 10^6$ cells in the tail vein or directly administered $1 \times 10^6$ cells into the bladder wall, as previously described (Dinney et al, 1995).

The growth of subcutaneous tumour was successively measured with a caliper. The greatest length of a tumour mass (a) and the width perpendicular to it (b) were measured every 5 days, and the tumour size was reported as a × b. Eight weeks after the injection of tumour cells in the tail vein, or 4 weeks after that in the bladder wall, the mice were sacrificed and the presence of tumour nodules was macroscopically examined in all abdominal and thoracic internal organs. The organs with tumour nodules were removed, and the number of surface tumour nodules was counted. The termination points of these studies were determined according to the preliminary experiments in order that tumour-related deaths may not occur before sacrifice.

### Statistical analysis

All of the data were evaluated by the Student's *t*-test. The levels of significance were set at $P < 0.05$.

### RESULTS

### Northern blot analysis of Bcl-2 mRNA levels among KoTCC-1 sublines

Expression levels of the Bcl-2 mRNA in KoTCC-1/P, KoTCC-1/C and the Bcl-2-transfected cell lines (KoTCC-1/BH1 to KoTCC-1/BH4) were analysed by Northern blotting. As shown in Figure 1, the Bcl-2 mRNA were highly expressed in four Bcl-2 transfected

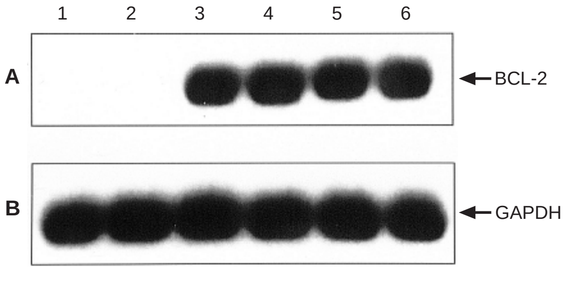

**Figure 1** Northern blot analysis of Bcl-2 mRNA in KoTCC-1 and Bcl-2-transfected cell lines. Total RNA was isolated and 30 μg of RNA was electrophoresed in 1.2% agarose gels and blotted onto a nylon membrane. The membrane was hybridized with $^{32}P$-labelled Bcl-2 cDNA specific for human Bcl-2 mRNA (**A**). After the removal of the radiolabelled probe, the membrane was rehybridized with GAPDH cDNA probe (**B**) to check the relative amounts of mRNA loaded on to the gel. Lane 1, KoTCC-1/P, parental cell line of KoTCC-1; Lane 2, KoTCC-1/C, vector only-transfected cell line; Lane 3, KoTCC-1/BH1; Lane 4, KoTCC-1/BH2; Lane 5, KoTCC-1/BH3; Lane 6, KoTCC-1/BH4. KoTCC-1/BH1 – KoTCC-1/BH4 are Bcl-2-transfected cell lines

© Cancer Research Campaign 1999

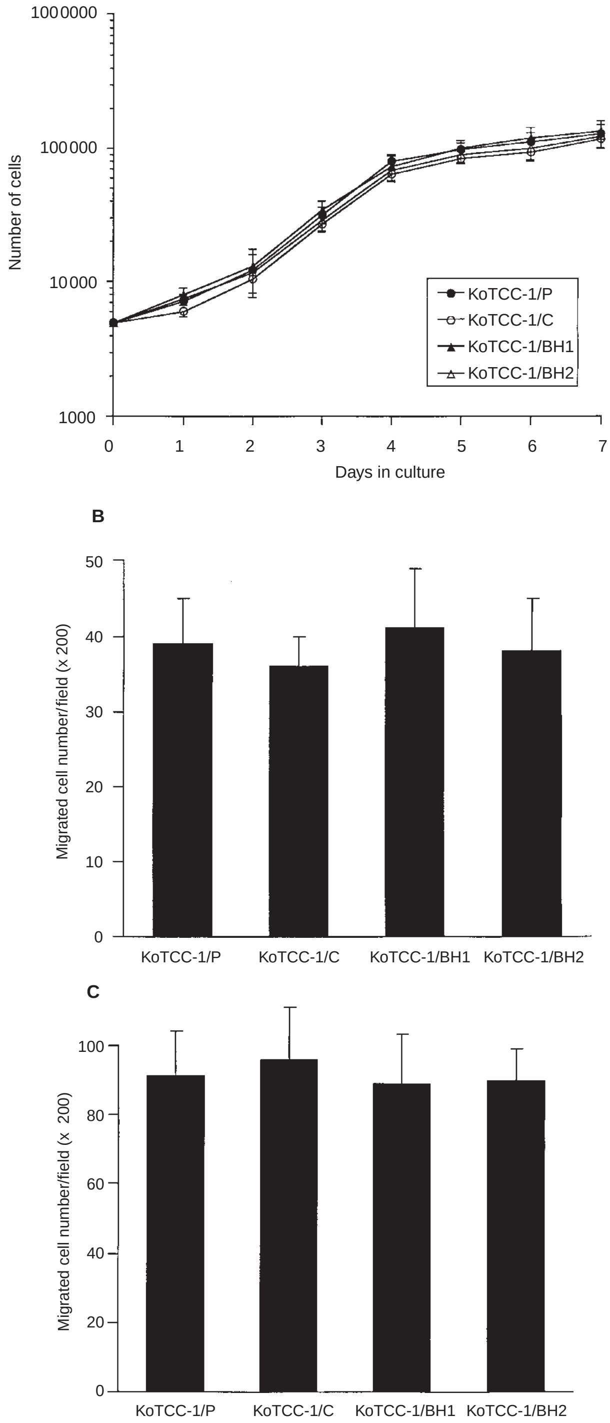

**Figure 2** (**A**) In vitro proliferation of KoTCC-1 and transfected clones of KoTCC-1. KoTCC-1/P cells, KoTCC-1 parental cell line; KoTCC-1/C cells transfected with control vector; KoTCC-1/BH1 cells transfected with the *bcl-2* gene (these cells expressed high levels of Bcl-2 protein); KoTCC-1/BH2 cells transfected with the *bcl-2* gene (these cells expressed nearly same levels of Bcl-2 as KoTCC-1/BH1). Five thousand cells of each cell line were seeded in 12-well plates. The cells were counted daily in triplicate. Bars represent standard deviations. (**B**) Measurement of in vitro invasive abilities of KoTCC-1 sublines. Each cell line was seeded at $1 \times 10^5$ per well in Boyden chambers. Chambers were incubated for 72 h in serum free DMEM/F-12, then numbers of cells that had migrated to lower surface of filters through reconstituted basement membrane Matrigel was counted at a 200 × magnification. Column and bars represent mean and standard deviation. (**C**) Measurement of cell motilities of KoTCC-1 sublines. Cell motility of each cell line was assessed by using Boyden chamber without Matrigel under the same condition as described above. Column and bars represent mean and standard deviation

**Table 1** Colonization of KoTCC-1 sublines upon limiting dilution

| Cell line[a] | Colony formation (%)[b] |
|---|---|
| KoTCC-1/P | 42.7 ± 5.2[c] |
| KoTCC-1/C | 44.8 ± 6.3 |
| KoTCC-1/BH1 | 91.7 ± 7.2[d] |
| KoTCC-1/BH2 | 88.5 ± 9.8[d] |

[a]Cells ($1 \times 10^2$) were diluted with RPMI plus 2% FCS and seeded into 96-well plates. The cells were cultured for 10 days. [b]Percentage of the wells in which cells grew was calculated. [c]Mean ± S.D. [d]The mean % of colony formation was significantly different from that of KoTCC-1/P and KoTCC-1/C at $P < 0.01$ (Student's *t*-test).

**Table 2** Cell viability of KoTCC-1 sublines in suspension

| | Surviving cells (%)[b] | |
|---|---|---|
| Cell line[a] | 24 h | 48 h |
| KoTCC-1/P | 52.4 ± 9.2[c] | 23.7 ± 7.7 |
| KoTCC-1/C | 56.5 ± 8.3 | 26.8 ± 8.3 |
| KoTCC-1/BH1 | 93.6 ± 8.8[d] | 59.3 ± 7.2[d] |
| KoTCC-1/BH2 | 90.5 ± 8.3[d] | 55.5 ± 6.8[d] |

[a]Cells ($5 \times 10^5$) were suspended in RPMI plus 2% FCS and subjected to 24 or 48 h floating. [b]Percentage of the viable cells normalized to the values obtained from control cells which were not subjected to floating. [c]Mean ± s.d. [d]The mean % of surviving cells was significantly different from that of KoTCC-1/P and KoTCC-1/C at $P < 0.01$ (Student's *t*-test).

clones at almost the same levels; in contrast, no detectable Bcl-2 mRNA was expressed in either KoTCC-1/P or KoTCC-1/C. The four Bcl-2-transfected clones showed almost the same results in the subsequent experiments; therefore, we hereafter report only the data of KoTCC-1/P, KoTCC-1/C, KoTCC-1/BH1 and KoTCC-1/BH2.

### Effects of Bcl-2 overexpression on in vitro malignant phenotypes of KoTCC-1 cells

Cell proliferation, invasive ability and cell motility are crucial factors for cancer metastasis; therefore, we initially determined whether cell proliferation, invasive ability and cell motility are altered by the overexpression of Bcl-2. There was no significant difference in cell proliferation in vitro among KoTCC-1/P, KoTCC-1/C, KoTCC-1/BH1 and KoTCC-1/BH2. Similar levels of the invasive potential and cell motility among KoTCC-1 sublines were also demonstrated by using trans-well culture chamber systems (Figure 2).

### In vitro anti-cell death activity of KoTCC-1 sublines

We then evaluated the relationship between the overexpression of Bcl-2 and the anti-cell death activity under anchorage-independent conditions among KoTCC-1 sublines by two different methods (i.e. limiting dilution assay and cell survival assay in suspension). As shown in Table 1, KoTCC-1/BH1 and KoTCC-1/BH2 exhibited significantly enhanced growth in limiting-dilution cultures

*© Cancer Research Campaign 1999*

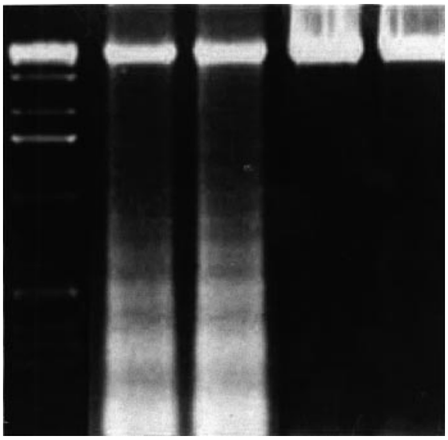

**Figure 3** DNA fragmentation analysis of KoTCC-1 sublines after 24 h of the floating assay. The isolated DNA was electrophoresed in a 2% agarose gel and stained with ethidium bromide. Lane 1, KoTCC-1/P; Lane 2, KoTCC-1/C; Lane 3, KoTCC-1/BH1; Lane 4, KoTCC-1/BH2. M = molecular weight markers (1 kb ladder; Life Technologies, Inc., Gaithersburg, MD, USA)

compared to KoTCC-1/P and KoTCC-1/C. The analysis of cell survival in the floating assay also demonstrated that the Bcl-2 transfectants had a powerful survival advantage in suspension compared to parental and control cells (Table 2). Furthermore, we performed DNA fragmentation analysis of each cell line subjected to 24-h floating in order to assess the apoptotic feature more definitely. The characteristic DNA ladders were observed in KoTCC-1/P and KoTCC-1/C but not in KoTCC-1/BH1 and KoTCC-1/BH2 (Figure 3).

### In vivo malignant potential of KoTCC-1 sublines

To examine the in vivo effects of Bcl-2 overexpression on tumour growth, $1 \times 10^6$ cells of each cell line were injected subcutaneously in the right flank of nude mice. There was no significant difference in tumour growth in vivo among KoTCC-1/P, KoTCC-1/C, KoTCC-1/BH1 and KoTCC-1/BH2 (Figure 4).

To investigate the effect of Bcl-2 overexpression on tumour progression, we injected $1 \times 10^6$ cells of each cell line into the tail vein or bladder wall. The mice were sacrificed 8 weeks after the intravenous injection, at which time we found that KoTCC-1/BH1 and KoTCC-1/BH2 had formed more than three times as many tumour nodules in lungs as had KoTCC-1/P and KoTCC-1/C (Table 3). The size of tumours in the lung were small (i.e. less than 2 mm), but could be clearly observed as a white nodule. Similarly, more marked differences were observed in tumour progression among KoTCC-1 sublines 4 weeks after the orthotopic implantation of each cell line; that is, as shown in Table 4, the incidence of retroperitoneal lymph node metastasis, mesenteric lymph node metastasis and haemorrhagic ascites in KoTCC-1/BH1 and KoTCC-1/BH2 were significantly higher than those in KoTCC-1/P and KoTCC-1/C. However, none of the primary tumours formed by KoTCC-1 sublines showed the symptoms of invasion into normal surrounding tissues, and there was no significant difference in the weights of the primary tumours among the KoTCC-1 sublines.

**Table 3** Lung colonization by KoTCC-1 sublines injected into the tail vein of nude mice

| Cell line[a] | No. of tumour nodules in the lung[b] |
|---|---|
| KoTCC-1/P | 10.4 ± 5.2[c] |
| KoTCC-1/C | 12.5 ± 4.1 |
| KoTCC-1/BH1 | 39.6 ± 9.5[d] |
| KoTCC-1/BH2 | 42.7 ± 8.4[d] |

[a]Cells ($1 \times 10^6$) were injected into the tail vein of nude mice. The mice were killed 8 weeks after injection. [b]The number of surface tumour nodules in the lung was determined. [c]Mean ± s.d. [d]The mean number of tumour nodules was significantly different from that of KoTCC-1/P and KoTCC-1/C at $P < 0.01$ (Student's *t*-test).

### DISCUSSION

The process of cancer metastasis consists of multiple steps and its regulation is extremely complicated (Fidler and Balch, 1987; Miyake et al, 1996). During metastasis, cancer cells detach from the primary origins in the body and must survive without adequate cell-to-cell or cell-to-substrate contact in the circulation (Takaoka et al, 1997; Nikiforov et al, 1997). Bcl-2 is the prototype of a novel class of oncogenes that contributes to neoplastic cell growth not by accelerating the rate of cellular proliferation but rather by enhancing the tumour cell survival through the inhibition of apop-

**Table 4** Tumour progression by KoTCC-1 sublines injected into the bladder wall of nude mice

| | Incidence of metastasis (%)[a] | | | |
|---|---|---|---|---|
| Cell line[c] | Retroperitoneal lymph node metastasis | Intra-abdominal lymph node metastasis | Incidence of haemorrhagic ascites (%)[b] | Weight of the primary tumour (mg) |
| KoTCC-1/P | 5/10 (50) | 2/10 (20) | 0/10 (0) | 32.2 ± 4.5[d] |
| KoTCC-1/C | 6/10 (60) | 2/10 (20) | 0/10 (0) | 34.1 ± 6.1 |
| KoTCC-1/BH1 | 10/10 (100)[e] | 7/10 (70)[e] | 5/10 (50)[e] | 30.9 ± 4.8 |
| KoTCC-1/BH2 | 10/10 (100)[e] | 8/10 (80)[e] | 5/10 (50)[e] | 33.6 ± 9.4 |

[a]Number of mice with tumour/number of injected mice. [b]Number of mice with haemorrhagic ascites/number of injected mice. [c]Cells ($1 \times 10^6$) were injected into the bladder wall. The mice were killed 4 weeks after injection. [d]Mean ± s.d. [e]The incidences of metastasis or haemorrhagic ascites were significantly different from that of KoTCC-1/P and KoTCC-1/C at $P < 0.01$ (Student's *t*-test).

© Cancer Research Campaign 1999

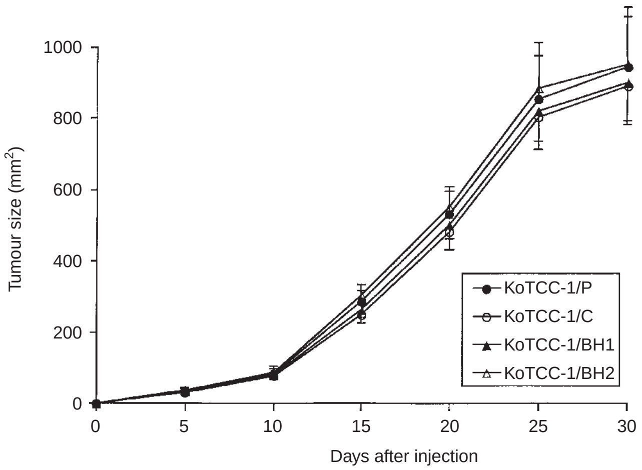

**Figure 4** Tumour growth in nude mice of the KoTCC-1 parental cell line (KoTCC-1/P), the control vector-transfected cell line (KoTCC-1/C) and clones of KoTCC-1-transfected Bcl-2 (KoTCC-1/BH1 and KoTCC-1/BH2). Nude mice were given $1 \times 10^6$ cells subcutaneously in the right flank on day 0. Tumour size was measured as the product of the greatest diameter multiplied by the perpendicular diameter. Bars represent standard deviations of tumour size tosis (Tsujimoto et al, 1985; Miyake et al, 1998*a*). Therefore, we hypothesize that Bcl-2 overexpression may contribute to prolong cancer cell survival against obstacles encountered in that process and ultimately thereby promote metastasis. We thus investigated the correlation of Bcl-2 overexpression with in vivo malignant potential by using the Bcl-2-transfected human bladder cancer cell lines (Miyake et al, 1998*a*).

Initially in order to rule out possibilities that in vitro malignant phenotypes of KoTCC-1 cells were enhanced by the overexpression of Bcl-2, we compared cell proliferation, invasive ability and cell motility among parental cells, control cells and the Bcl-2 transfectants. As expected, no significant differences were observed among these cell lines in the in vitro phenotypes we examined. We then investigated the effect of Bcl-2 overexpression on the anti-cell death activity under anchorage-independent conditions by limiting dilution assay and cell survival assay in suspension. In both assays, the Bcl-2 transfectants exhibited significantly superior viability in comparison with control and parental cells. These in vitro findings strongly suggest that Bcl-2 may give enhanced ability to survive under anchorage-independent conditions like those cancer cells encounter in circulation during metastatic formation.

We next demonstrated that the Bcl-2 transfectants showed more extensive malignant progression than parental and control cells by using the intravenous and orthotopic tumour cell injection models. These data revealed that the in vivo tumour progression of these cell lines was closely correlated with their in vitro anti-cell death activity under anchorage-independent conditions. In addition, there were no significant differences in tumour growth among these cell lines, when they were implanted in either ectopic (subcutaneous) or orthotopic (bladder) organs. We also showed that no remarkable differences in in situ apoptotic features among subcutaneous and bladder tumours formed by these cell lines by the terminal deoxynucleotidyl transferase-mediated deoxyuridine 5′-triphosphate nick and labelling (TUNEL) staining (data not shown). Considering these findings, we conclude that the phenomenon of the enhanced anti-apoptotic activity may not be entirely responsible for the enhanced in vivo malignant potential of the Bcl-2-transfected cells, but there is no doubt that anti-cell death activity is involved in promoting the tumour progression in vivo.

Recent studies have revealed that several molecules, including p53 (Nikiforov et al, 1997), DAP kinase (Inbal et al, 1997), BAG-1 (Takaoka et al, 1997) and CC3 (Shtivelman, 1997), play crucial roles in metastatic process via the regulation of cell survival under anchorage-independent conditions. These findings suggest that Bcl-2 alone is not likely to reflect anti-apoptotic activity in the tumour cells. Furthermore, we consider it important to examine the effect of Bcl-2 on the expression levels of specific inhibitor and promoter genes of metastasis to address a possible mechanism for enhanced metastatic potential of the Bcl-2-transfected cells more completely. We also think it important to assess the effect of Bcl-2 on tumour cells in the circulation and early post-extravasation phase in order to explore the actual apoptotic feature under anchorage-independent conditions and provide clues underlying the potential therapeutic strategy against early stage of metastasis. For this purpose, as several previous studies suggested (Crowley et al, 1993; Tsuchiya et al, 1993), it seems to be an attractive approach to use easily detectable markers, such as radio isotope, luciferase and chloramphenicol acetyltransferase.

In conclusion, our present results indicating enhancement of malignant potential of bladder cancer cells in vivo by overexpression of Bcl-2 protein reveals a crucial role of anti-apoptotic activity during tumour progression. Therefore, the expression of Bcl-2 protein in bladder cancer cells may be a possible candidate as a marker predicting development of bladder cancer.

### REFERENCES

Albini A, Iwamoto Y, Kleinman HK, Martin GR, Kozlowski JM and McEwan RN (1987) A rapid in vitro assay for quantitating the invasive potential of tumor cells. *Cancer Res* **47**: 3239–3245

Crowley CW, Cohen RL, Lucas BK, Liu G, Shuman MA and Levinson AD (1993) Prevention of metastasis by inhibition of the urokinase receptor. *Proc Natl Acad Sci USA* **90**: 5021–5025

Dinney, CPN, Fishbeck R, Singh RK, Eve B, Pathak S, Brown N, Xie B, Fan D, Bucana CD, Fidler IJ and Killion JJ (1995) Isolation and characterization of metastatic variants from human transitional cell carcinoma passaged by orthotopic implantation in athymic nude mice. *J Urol* **154**: 1532–1538

Ellis RE, Yuan JY and Horvitz HR (1991) Mechanism and functions of cell death. *Ann Rev Cell Biol* **7**: 663–698

Fidler IJ and Balch CM (1987) The biology of cancer metastasis and implication for therapy. *Curr Prob Surg* **24**: 129–209

Gallo O, Boddi V, Calzolari A, Simonetti L, Trovati M and Bianchi S (1996) bcl-2 protein expression correlates with recurrence and survival in early stage head and neck cancer treated by radiotherapy. *Clin Cancer Res* **2**: 261–267

Inbal B, Cohen O, Polak-Charcon S, Kopolovic J, Vadai E, Einsenbach L and Kimchi A (1997) DAP kinase links the control of apoptosis to metastasis. *Nature* **390**: 180–184

King ED, Matteson J, Jacobs SC and Kyprianou N (1996) Incidence of apoptosis, cell proliferation and bcl-2 expression in transitional cell carcinoma of the bladder: association with tumor progression. *J Urol* **155**: 316–320

Miyake H, Hara I, Yoshimura K, Eto H, Arakawa S, Wada S, Chihara K and Kamidono S (1996) Introduction of basic fibroblast growth factor gene into mouse renal cell carcinoma cell line enhances its metastatic potential. *Cancer Res* **56**: 2440–2445

Miyake H, Yoshimura K, Hara I, Eto H, Arakawa S and Kamidono S (1997) Basic fibroblast growth factor regulates matrix metalloproteinases production and in vitro invasiveness in human bladder cancer cell lines. *J Urol* **157**: 2351–2355

© Cancer Research Campaign 1999

Miyake H, Hanada N, Nakamura H, Kagawa S, Fujiwara T, Hara I, Eto H, Gohji K, Arakawa S, Kamidono S and Saya H (1998a) Overexpression of Bcl-2 in bladder cancer cells inhibits apoptosis induced by cisplatin and adenoviral-mediated p53 gene transfer. *Oncogene* **16**: 933–943

Miyake H, Hara I, Gohji K, Yoshimura K, Arakawa S and Kamidono S (1998b) Expression of basic fibroblast growth factor is associated with resistance to cisplatin in a human bladder cancer cell line. *Cancer Lett* **123**: 121–126

Nikiforov MA, Hagen K, Ossovskaya VS, Connor TMF, Lowe SW, Deichman GI and Gudkov A (1997) p53 modulation of anchorage independent growth and experimental metastasis. *Oncogene* **13**: 1709–1719

Shtivelman E (1997) A link between metastasis and resistance to apoptosis of variant small cell lung carcinoma. *Oncogene* **14**: 2167–2173

Takaoka A, Adachi M, Okuda H, Sato S, Yawata A, Hinoda Y, Takayama S, Reed JC and Imai K (1997) Anti-cell death activity promotes pulmonary metastasis of melanoma cells. *Oncogene* **14**: 2971–2977

Trasher JB and Crawford ED (1993) Current management of invasive and metastatic transitional cell carcinoma of the bladder. *J Urol* **149**: 957–961

Tsuchiya Y, Sato H, Endo Y, Okada Y, Mai M, Sasaki T and Seiki M (1993) Tissue inhibitor of metalloproteinase 1 is a negative regulator of the metastatic ability of human gastric cancer cell line, KKLS, in the chick embryo. *Cancer Res* **53**: 1397–1402

Tsujimoto Y, Gorham J, Cossman J, Jaffe E and Croce CM (1985) The t(14;18) chromosome translocations involved in B-cell neoplasms result from mistakes in VDJ jointing. *Science* **229**: 1390–1393

*© Cancer Research Campaign 1999*
